# Pitfalls in Emergency Department Focused Bedside Sonography of First Trimester Pregnancy

**DOI:** 10.1155/2013/982318

**Published:** 2013-08-12

**Authors:** Kerri Layman, Michael Antonis, Jonathan E. Davis

**Affiliations:** Department of Emergency Medicine, MedStar Georgetown University Hospital, MedStar Washington Hospital Center, Georgetown University School of Medicine, Ground Floor, CCC Building, 3800 Reservoir Road, NW, Washington, DC 20007, USA

## Abstract

*Background*. Bedside sonography performed by emergency physicians is frequently utilized for real-time clinical decision-making in the emergency department (ED) setting. This includes the sonographic evaluation of pain or bleeding in the first trimester of pregnancy. The detection of intrauterine pregnancy (IUP) or life-threatening conditions, including ectopic pregnancy, is critical. *Objectives*. This paper will review several important pearls and avoidable pitfalls of this diagnostic modality by brief presentation of illustrative cases followed by discussion of key principles. *Case Reports*. Three patients evaluated in the ED for bleeding or pain occurring during the first trimester of pregnancy will be presented. *Conclusions*. When conducting emergency bedside ultrasound for the evaluation of first trimester pregnancy, it is important to avoid common pitfalls that can place your patient at risk.

## 1. Introduction

In recent years, studies have demonstrated that emergency physicians (EPs) can competently perform focused bedside sonography for the evaluation of first trimester pregnancy in the emergency department (ED) [1, 2]. Indeed, EP utilization of ultrasound in first trimester pregnancy is becoming increasingly more common and accepted within emergency care. The American College of Emergency Physicians (ACEP) lists emergency ultrasound in pregnancy as a core area of ultrasound proficiency for the emergency medicine (EM) specialist. In addition, all EM residents are now required to become facile with bedside ultrasound [3]. The detection of potentially life-threatening problems in early pregnancy, particularly ectopic pregnancy, is a fundamental skill [4, 5]. Given the widespread use of this modality, we seek to point out several important and avoidable pitfalls in bedside sonography for first trimester pregnancy through use of representative cases. Important pearls and strategies to avoid these pitfalls are highlighted.

## 2. Case Presentations

### 2.1. Case 1: *β*hCG Level below the Discriminatory Zone

**Figure 1:**
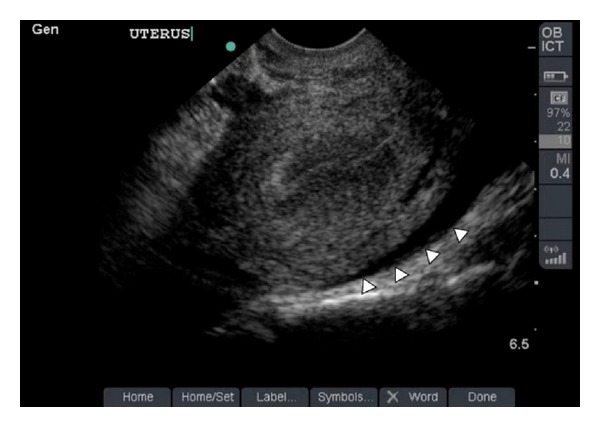
Utilizing a 5–8 MHz Sonosite intracavitary transducer, an empty uterus is noted. Posterior to the uterus, there is a region of hypoechoic fluid visualized (arrowheads).

A 27-year-old female, Gravida 2, Para 1, presented to the ED following delivery by cesarean section four months priorly, with absence of menstruation since the time of delivery. She presented with severe sharp cramping lower abdominal pain of 18-hour duration. Her vital signs were as follows: temperature 37 degrees centigrade, blood pressure 133/84 millimeters of mercury, heart rate of 156 beats per minute, and a normal respiratory rate and room air oxygen saturation. A urine pregnancy test was positive. The serum quantitative beta human chorionic gonadotropin (*β*hCG) level was 726 international units per liter (IU/L). Focused, screening bedside transabdominal ultrasound followed by transvaginal ultrasound examination to evaluate for IUP performed by the treating EP revealed free fluid in the abdomen and the absence of a gestational sac in the uterus (Figure 1). Based on the finding of a positive *β*hCG test and free intraperitoneal fluid on sonogram, the gynecology service was emergently consulted. Despite a quantitative *β*hCG level below the “discriminatory zone,” the patient underwent emergent laparoscopic right salpingectomy for ectopic pregnancy with evacuation of 500 mL of clotted blood from the peritoneum.

### 2.2. Case 2: *β*hCG Level near or above the Discriminatory Zone and an Empty Uterus

A 26-year-old female, Gravida 4, Para 2, presented to the ED with suprapubic cramping and dysuria. Her last menses was one month priorly. Her vital signs were all normal. A urine pregnancy test was positive. Urine dipstick revealed moderate blood and small leukocyte esterase. An EP performed focused, bedside emergency transabdominal followed by transvaginal ultrasound examination which was nondiagnostic; no IUP was identified. The quantitative *β*hCG level was 1,484 IU/L. Given the *β*hCG level just below the “discriminatory zone” and a concomitant diagnosis of possible urinary tract infection (UTI), the patient was treated with antimicrobials for UTI in pregnancy and discharged home. Given the non-diagnostic ultrasound, the patient was instructed to return to the ED or her gynecologist in 2-3 days for a repeat *β*hCG level determination and a repeat ultrasound examination. 

**Figure 2:**
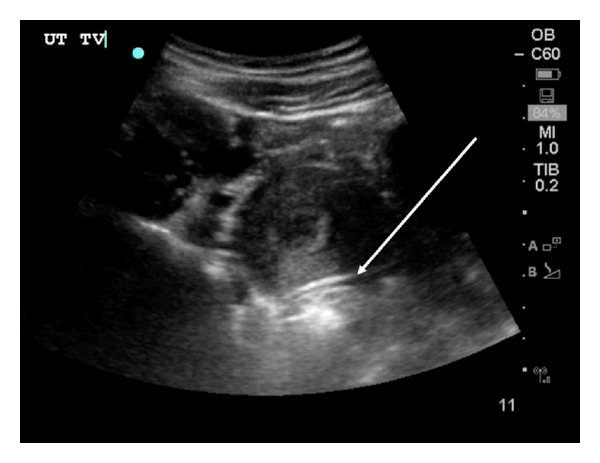
Utilizing a Sonosite C60 2–5 MHz curvilinear transducer, this transabdominal ultrasound image displays an empty uterus with a thin sliver of hypoechoic fluid posterior to the uterine wall (arrow).

**Figure 3:**
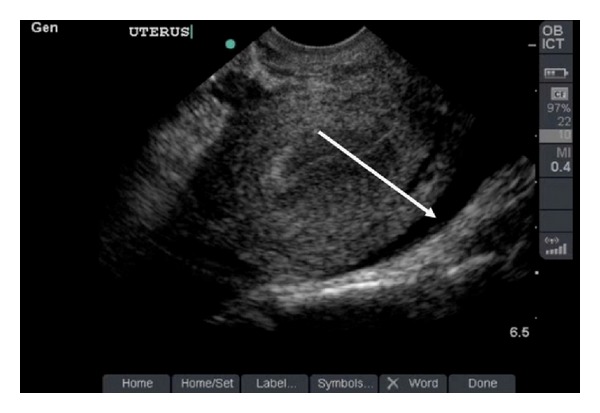
Pelvic ultrasound demonstrates the same hypoechoic fluid collection posterior to the uterine wall seen on transabdominal ultrasound in Figure 2.

Following patient discharge, free intraperitoneal fluid in the cul de sac was identified on routine ED ultrasound quality improvement review (Figures 2 and 3). This fluid was not fully appreciated by the treating provider at the time of the initial study. The patient did not follow up as instructed but eight days later returned to the ED complaining of right lower quadrant abdominal pain. Her vital signs were again normal. During the return visit, the serum *β*hCG level had risen to 5,775 IU/L. Emergency bedside transabdominal and transvaginal ultrasound revealed no IUP. The gynecology service was consulted, and further diagnostic transvaginal ultrasound imaging by the gynecology service revealed a 5.5 cm right-sided mass suspicious for an ectopic pregnancy. The patient underwent a laparoscopic right salpingectomy with a postoperative course complicated by pelvic hematoma.

### 2.3. Case 3: The EP Sonographer's Unique Vantage Point in Synthesizing Clinical, Laboratory, and Imaging Data

**Figure 4:**
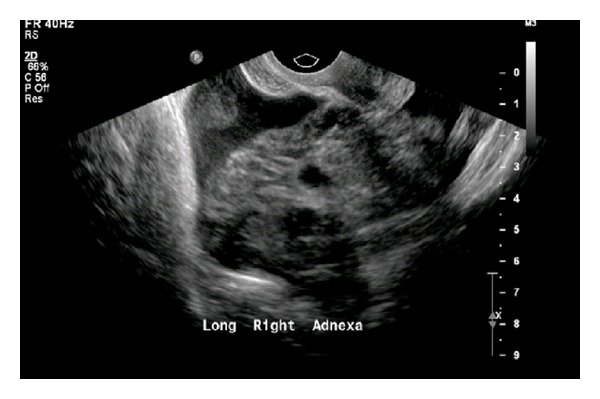
Pelvic ultrasound (long view, right adnexa) demonstrates a fluid collection of mixed echogenic and anechoic material surrounding the right ovary.

A 23-year-old female, Gravida 3, Para 2, presented complaining of cramping pelvic pain and vaginal bleeding. Her last menses was one month priorly. A urine pregnancy test was positive. The quantitative *β*hCG level was 398 IU/L. Focused, bedside transabdominal and transvaginal ultrasound performed by the EP was suspicious for ectopic pregnancy based on the lack of a recognized IUP and focal pain and tenderness in the right lower quadrant. Per local protocol, a repeat ultrasound examination was immediately performed in the radiology suite. The radiologist determined that there was an abnormal fluid collection around the right ovary most consistent with a hematoma (Figure 4). The study was formally interpreted as no definite adnexal mass and no definite IUP. The patient was further evaluated by the gynecology service due to the EP's continued suspicion for ectopic pregnancy. She was ultimately discharged home following specialty consultation with instructions to return in 2 days for repeat serum *β*hCG level and follow-up ultrasound examination.

**Figure 5:**
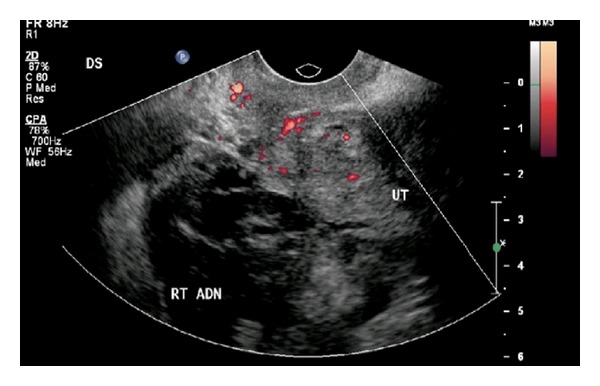
Pelvic ultrasound demonstrates a right adnexal mass (labeled RT ADN) adjacent to the uterus (labeled UT) with surrounding hypoechoic fluid suspicious for ectopic pregnancy.

The patient returned to the ED within 48 hours, complaining of worsening pain, vaginal bleeding, and lightheadedness. Vital signs revealed a heart rate of 121 beats per minute and a blood pressure of 146/74 millimeters of mercury. The *β*hCG level had risen to 455 IU/L, and transvaginal ultrasound (bedside and in the radiology suite) performed during the repeat visit confirmed a right-sided ectopic pregnancy with associated free intraperitoneal fluid (Figure 5). The patient initially underwent a laparoscopic right salpingectomy, which required intraoperative conversion to open laparotomy because of the presence of adhesions between the uterus and anterior abdominal wall, as well as a significant number of blood clots obscuring the view of the camera. The patient ultimately had an uneventful recovery.

## 3. Discussion

### 3.1. Pitfall 1: Failure to Obtain a Diagnostic Ultrasound Imaging Study When the *β*hCG Quantitative Value Is below the Discriminatory Zone

The first case illustrates a common misperception with first trimester emergency ultrasound. That is, an ectopic pregnancy is unlikely to be diagnosed by bedside ultrasound when the *β*hCG is below the “discriminatory zone.” Published guidelines regarding the “discriminatory zone” often lead to confusion in the approach to patients with early pregnancy. The discriminatory zone is defined as the level of *β*HCG above which an IUP can be reliably detected by ultrasound [6]. This level is frequently defined as 1,500 IU/L by transvaginal ultrasound and 6,500 IU/L by transabdominal ultrasound [7–9]. Though the discriminatory zone concept was developed when only transabdominal ultrasound was standard, it is now routine to obtain a transvaginal pelvic ultrasound to evaluate IUP in early pregnancy. The discriminatory zone range for transvaginal ultrasound varies among practitioners and institutions but typically falls between 1,500 IU/L and 3,000 IU/L [10]. A more conservative approach is to use the lower end of this range (1,500 IU/L) in emergency clinical decision-making. Ectopic pregnancy, however, may be detected at *β*hCG levels well below the lower end of the discriminatory zone range [11]. In comparing 6 different strategies for diagnosing IUP, the strategy of ultrasound for all, as opposed to ultrasound for only those with a quantitative *β*hCG above the discriminatory zone, was the most sensitive [12]. Indeed, a study conducted by Condous and his colleagues showed that use of the 1,500 IU/L level as a cutoff is only 15% sensitive in detecting ectopic pregnancy [13]. Further, a recent ED-based study again revealed the lack of ability of *β*hCG level to assist in determining intrauterine versus ectopic pregnancy [14]. Therefore, it is important for the EP to consider the diagnosis of ectopic pregnancy with pain or bleeding and consider diagnostic ultrasound even if the *β*hCG level falls below the discriminatory zone threshold value.

### 3.2. Pitfall 2: Failure to Obtain Additional Diagnostic Imaging or Specialty Consultation When Faced with a *β*hCG Quantitative Level near or above the Discriminatory Zone in the Setting of an Empty Uterus

The second case illustrates another common pitfall. The American College of Emergency Physicians (ACEP) guidelines state that the primary goal of obstetric ultrasound in the ED is to determine if an IUP is present [3]. Further diagnosis by bedside ultrasound when an IUP cannot be identified has lower sensitivity rates. Since the EP recognized that there was no IUP present in the setting of a *β*HCG level near the discriminatory zone, further radiologic ultrasound on the initial visit would have been appropriate. In addition, a study of women at risk for ectopic pregnancy confirms that the presence of echogenic fluid is a significant risk factor for the ultimate diagnosis of ectopic pregnancy [15]. Whether or not the treating EP appreciated the small amount of free fluid detected during the quality improvement image review, the lack of an IUP despite a *β*HCG near or above the discriminatory zone should have prompted consideration of further diagnostic imaging. The bottom line is that it is prudent for the EP to consider this scenario (*β*hCG near or above the discriminatory zone accompanied by lack of a defined IUP on ultrasound examination of the uterus) to be high risk for ectopic pregnancy until proven otherwise, prompting further diagnostic imaging or specialty consultant engagement to aid in decision-making.

### 3.3. Pitfall 3: Failure to Trust One's Judgment as the Treating Clinician When Synthesizing Historical, Examination, and Diagnostic Imaging and Laboratory Data

The final case illustrates the potential pitfall of not trusting your instincts as the clinician sonographer present at the bedside. The ability of EPs to make decisions based on bedside ultrasound of first trimester pregnancy has been illustrated in numerous studies [5, 15, 16]. One such study revealed a 96% concordance rate with radiologic interpretation [17]. In addition, the EP has the added benefit of being at the patients' bedside. This provides the unique vantage point of synthesizing historical, examination, laboratory, and imaging findings in the overall clinical context. When the picture just does not seem to fit, consider further diagnostic imaging or specialty consultation. In the case presented, these additional steps were taken, and the diagnosis was ultimately made at the time of follow-up. This underscores the importance of detailing and documenting strict follow-up precautions, including indications to return to the ED and the need for frequent serial reevaluations.

## 4. Conclusion

When conducting emergency bedside ultrasound for the evaluation of first trimester pregnancy, it is important to avoid common pitfalls that can place your patient at risk. Primarily, all patients with a positive pregnancy test should undergo ultrasound evaluation regardless of the *β*HCG level. Second, if a definitive IUP is not visualized when the *β*hCG level is near or above the discriminatory zone, maintain a very low threshold for further diagnostic imaging and, in some cases, specialty consultation. Finally, trust your judgment. EPs have a unique vantage point in being able to synthesize historical, examination, laboratory, and imaging data, as well as consideration of alternate diagnoses. If in doubt, review your findings with your consultants to come up with the most likely diagnosis and ensure timely follow-up evaluation for your patient.
